# Asymmetry in the burial of hydrophobic residues along the histone chains of Eukarya, Archaea and a transcription factor

**DOI:** 10.1186/1472-6807-5-20

**Published:** 2005-10-21

**Authors:** B David Silverman

**Affiliations:** 1 IBM Thomas J. Watson Research Center P. O. Box 218, Yorktown Heights, NY 10598 USA

## Abstract

**Background:**

The histone fold is a common structural motif of proteins involved in the chromatin packaging of DNA and in transcription regulation. This single chain fold is stabilized by either homo- or hetero-dimer formation in archaea and eukarya. X-ray structures at atomic resolution have shown the eukaryotic nucleosome core particle to consist of a central tetramer of two bound H3-H4 dimers flanked by two H2A-H2B dimers. The c-terminal region of the H3 histone fold involved in coupling the two eukaryotic dimers of the tetramer, through a four-fold helical bundle, had previously been shown to be a region of reduced burial of hydrophobic residues within the dimers, and thereby provide a rationale for the observed reduced stability of the H3-H4 dimer compared with that of the H2A-H2B dimer. Furthermore, comparison between eukaryal and archaeal histones had suggested that this asymmetry in the distribution of hydrophobic residues along the H3 histone chains could be due to selective evolution that enhanced the coupling between the eukaryotic dimers of the tetramer.

**Results and discussion:**

The present work describes calculations utilizing the X-ray structures at atomic resolution of a hyperthermophile from *Methanopyrus kandleri *(HMk) and a eukaryotic transcription factor from *Drosophila melanogaster *(DRm), that are structurally homologous to the eukaryotic (H3-H4)_2 _tetramer. The results for several other related structures are also described. Reduced burial of hydrophobic residues, at the homologous H3 c-terminal regions of these structures, is found to parallel the burial at the c-terminal regions of the H3 histones and is, thereby, expected to affect dimer stability and the processes involving histone structural rearrangement. Significantly different sequence homology between the two histones of the HMk doublet with other archaeal sequences is observed, and how this might have occurred during selection to enhance tetramer stability is described.

## Background

The histone fold [1] is a commonly conserved structural motif found in proteins that interact with DNA [2-6]. This monomeric fold consists of a helix-strand-helix motif stabilized by dimer formation. X-ray structures at atomic resolution, in either the absence [1] or presence of DNA [7], have shown the octamer of the eukaryotic nucleosome to be composed of histone dimers in a "handshake conformation" [1], with the H3-H4 dimers coupled forming a central tetramer flanked by two H2A-H2B dimers.

The dimers of the tetramer are bound by a tenuous four-fold helical bundle that involves residues over the c-terminal regions of the H3 histone folds. Consequently, side chain orientations in these regions while contributing to tetramer stability do not contribute to the stability of the dimers in the tetramer conformation by optimally burying residues within the dimer interiors. Such orientations, if comparably maintained in the isolated dimers would, therefore, reduce dimer stability relative to that of dimers that are not required to perform such dual role of hydrophobic burial, namely, burial with respect to the interface between dimers as well as with respect to the dimer interiors. This has been proposed [8] as a rationale for the observed [9] reduced stability of the H3-H4 dimer compared with that of the H2A-H2B dimer.

X-ray structures at atomic resolution of an ancestral nuclear protein histone of the hyperthermophile, HMk, from *Methanopyrus kandleri *[10] and of the amino-terminal portions of the TFIID transcription factor TATA box-binding associated factors (TAF_II_s), dTAF_II_42 and dTAF_II_62, from *Drosophila*, DRm [11], have been found to be structurally homologous to the eukaryotic (H3-H4)_2 _tetramer. Since the unusual fold of the hyperthermophile contains two histone fold motifs arranged in tandem within a single chain [10,12], its structure, homologous to the eukaryotic tetramer, is a dimer. The coupling of the HMk monomers and TFIID dimers that yield the higher-order dimeric and tetrameric structures, respectively, involves a four-fold helical bundle as does the (H3-H4)_2 _tetramer. Since the c-terminal region of the H3 histone fold involved in coupling the two eukaryotic nucleosomal dimers of the tetramer through the four-fold helical bundle had previously been shown [8] to be a region of reduced burial of hydrophobic residues within the H3-H4 dimers, it is of interest to investigate the burial of hydrophobic residues over the homologous c-terminal regions of the HMk monomer and DRm dimer. Reduced stability of these lower-order structures, due to the burial of hydrophobic residues within the four-fold helical bundles, holds implications for dynamical processes involving DNA as well for nucleosome assembly.

Calculations have, therefore, been performed for structures of the HMk monomer and DRm dimer, as performed previously for archaeal methanogens and eukaryotic nucleosomal dimers [8]. An interesting pattern emerges. All chains involved in the coupling of the lower-order structures, e.g. the HMk monomers and DRm dimers that yield dimers and tetramers, respectively, exhibit a difference in the burial of hydrophobic residues along their length. Reduced burial in the lower-order structures arises from the region of the coupling, namely, the region of the four-fold helical bundle. While these results are consistent with the difference in burial of hydrophobic residues from the n- to c-terminal regions of the H3 histone chains of the nucleosome, the magnitudes of the burial over the homologous c-terminal regions of the HMk and DRm chains are found to be less than that of the H3 magnitudes. On the other hand, all eukaryotic chains that are not involved in the helical coupling yielding the tetramers, do not exhibit such difference in hydrophobic burial of residues along their length.

From a structural perspective, one expects the H3 histone to play a key role in organizing the nucleosome. From an evolutionary perspective, the n-terminal histone of HMk is clearly cast in that role [13]. While the archaeal n-terminal histone of HMk is found to exhibit little or no sequence homology with other archaeal histones, BLAST2 as well as PSI-BLAST searches, show the c-terminal histone, the histone structurally homologous with the H4 eukaryotic histone, to exhibit significant homology with other archaeal histones. How selection, involving the n-terminal histone of HMk, to enhance tetramer stability could have evolved within the context of such dichotomy is described.

Early recognition of the homology between the sequences of the *Drosophila *transcription factor and the eukaryotic H3 and H4 histones had suggested [14] the presence of a histone octamer-like TAF complex within TFIID that would interact with DNA in a manner similar to that of the histone octamer. Subsequent investigations [15,16] have provided chromatographic evidence suggesting the presence of a TAF octamer within the TFIID initiation complex. Comparisons of the structural and sequence homology between the tetramers of HMk, DRm and the eukaryotic H3 and H4 histones show, as expected, that the structures and sequences of species belonging to the same domain align more closely than those belonging to different domains. While the HMk dimer appears to be structurally homologous to the eukaryotic tetramer, a detailed analysis highlights differences.

## Methods

Calculations have been performed utilizing the PDB structures of the histone monomer and dimer of the ancestral hyperthermophile from *Methanopyrus kandleri*, HMk [10] (PDB id 1F1E) and the eukaryotic transcription TATA box-binding associated factors (TAF_II_s) dTAF_II_42 and dTAF_II_62 from *Drosophila melanogaster*, DRm [11] (PDB id 1TAF), respectively. Only the structural segments with histone fold motifs, i.e., the helix-strand-helix-strand-helix substructure segments as defined by the PDB files are extracted and used in the calculations. The HMk single chain is thus clipped in the middle and the HMk structure then appears as a histone dimer. The histone nearest the n-terminal end of the HMk monomer will be simply called the "first histone". The histone nearest the c-terminal end of the monomer will be called the "second histone". Consequently the n-terminal region of the HMk monomer may be referred to as the n-terminal region of the first histone and the c-terminal region of the HMk monomer referred to as the c-terminal region of the second histone. Dependent upon the context of discussion, the HMk monomer may be referred to as an HMk dimer and the dimer referred to as a tetramer. This terminology supplements that used previously [10] enabling a more unequivocal and parallel description of the histone homologies between HMk and other species. The "c-terminal region" of the first HMk histone is then the region involved in the four-fold helical coupling that binds the HMk dimers of the tetramer. This region is structurally homologous with the c-terminal region of the H3 eukaryotic histone. One might have been tempted to label the two segments of HMk, domain1 and domain2. Since this protein nomenclature is usually reserved for units that appear to fold independently and the histone fold is stabilized by dimerization, we have not used this terminology. The dimer of the eukaryotic *Drosophila melanogaster *transcription factors, dTAF_II_42 and dTAF_II_62 will be referred to as the DRm dimer. Consistent with previous usage [10], the hyperthermophile *Methanopyrus kandleri *will be referred to as HMk. The HMk dimer (tetramer) and the DRm tetramer may be referred to as the higher-order structures, collectively.

Calculations have also been performed on the archaeal histone dimer, HPha from *Pyrococcus horikoshii *[17], and on two other histone-like transcription associated factor (TAF) dimeric structures. One is from the hetero-trimeric transcription factor, NF-Y [18] (PDB id 1N1J), and the other is from the ternary complex of the negative cofactor 2, NC2 [19] (PDB id 1JFI)). The results of calculations performed on these structures for which structural homology with the eukaryotic tetramer is not expected, are useful for comparison with the results obtained for the HMk and DRm structures.

The present calculations, which provide a measure of residue distance from the interior of the dimers, and consequently, correlation coefficients between these distances and residue hydrophobicities, are based upon the residue side-chain locations. The center-of-geometry of the *i*th residue, or residue centroid, , is calculated with inclusion of only the backbone α – carbon atom and exclusion of the hydrogen atoms. This distribution of points in three-dimensional space enables calculation of the geometric center of the distribution, , namely, the centroid of all residue side-chain centroids of the protein. This will be called the "center-of-the-protein".



n is the total number of residues.

An ellipsoidal characterization of protein shape is obtained as follows.

A second rank geometric tensor



with , the unit dyadic, is diagonalized to provide the moments-of-geometry, *g*_1_, *g*_2_, and *g*_3_. These moments are the moments-of-inertia of a discrete distribution of points of unit mass. The moments provide an ellipsoidal characterization of protein shape.



*x*_*p*_, *y*_*p*_, *z*_*p*_, are coordinates in the frame of the principal axes with the centroid of the structure as origin. If the magnitudes are ordered as,

*g*_1 _<*g*_2 _<*g*_3 _    [4]

the major principal axis is of length, (*d*^2^/*g*_1_)^1/2^.

Each *i*th residue at location, *x*_*ip*_, *y*_*ip*_, *z*_*ip*_, in the frame of the principal axis, resides on an ellipsoid with the length of its major principal axis equal to, , namely,



For a compact globular protein, the residue associated with the largest *d*_*i *_specifies the ellipsoid that defines a presumed "protein surface". Residues with the same *d*_*i*_, namely, residues residing on the same ellipsoid are at the same radial fractional distance from the center-of-the-protein to the protein ellipsoidal surface.

Rewriting equation 5 as:



with 

enables  to be used as a measure of the radial fractional distance of the *i*th residue from the center-of-the-protein to the protein surface.

This distance, which will be called the ellipsoidal distance, is used in the calculations. It is just the value of the principal major axis of the ellipsoid upon which the residue centroid is found. It has been shown to correlate more closely with residue solvent accessibility than the radial distance from the center-of-the-protein to the residue centroid [20].

The scale of residue hydrophobicity chosen is that of Neumaier [21]. This scale provided greater correlation between residue hydrophobicity and ellipsoidal distance than a number of other scales initially considered. Calculations are only performed on the histone folds when in the conformation of the dimer. Consequently the residue distances obtained from the calculations are distances from the center of the dimer. This enables an examination of how the burial of residue hydrophobicity within the four-fold helical bundle trades-off against such burial within the interior of the dimer. The degree of such trade-off is quantitatively mirrored by the correlation coefficient between residue distances from the dimer interior and residue hydrophobicities.

A number of web based programs, i.e., PSI-BLAST [22], BLAST2 [23], CE [24] and CONSURF [25] have been used in examining the homology between the histones of HMk and DRm with other archaeal and eukaryal sequences.

## Results and discussion

**Figure 1:**
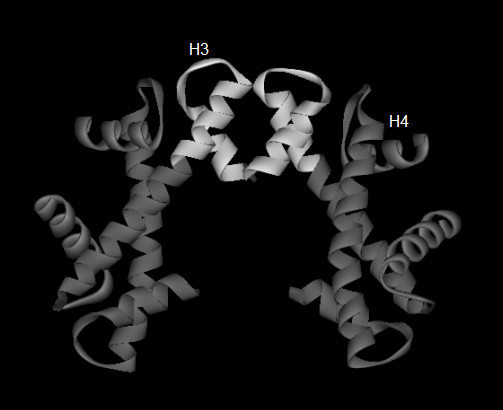
Highlighted region of the four-fold helical coupling of the (H3-H4)_2 _tetramer.

Tables 1 and 2 list the correlation coefficients previously obtained [8] between residue distances from the dimer interior and residue hydrophobicity of the amino acids of the H3, H4, H2A, and H2B histone chains. Histones from the *Xenopus laevis *[26] (PDB id 1KX5), *Gallus gallus *[27] (PDB id 1EQZ) and *Saccharomyces cerevisae *[28] (PDB id 1ID3) species had been used in the calculations. The correlation coefficients are provided for the entire histone chain as well as for the n- and c-terminal halves of the chain. Figure 1 highlights the c-terminal regions of the A and E chains of the H3 histones of the 1KX5 structure involved in the four-fold helical coupling that binds the dimers of the tetramer. Residues in these regions comprise a majority of the residues of the c-terminal halves of the chains. Table 1 shows significant reduction in correlation coefficient between amino acid distance from the center of the dimer and hydrophobicity over the c-terminal halves of the H3 chains of the three species compared with their n-terminal halves. The c-terminal regions of the H3 histone folds are regions of reduced burial of hydrophobic residues within the dimers and thereby provide a rationale for the observed reduced stability of the H3-H4 dimer compared with that of the H2A-H2B dimer [9]. Tables 1 and 2 also show that histones not involved in the four-fold helical coupling yielding the higher order tetrameric structures, namely, H4, H2A, and H2B, do not exhibit the extent of differential burial of hydrophobic residues over their chain-lengths as found for the H3 histones.

Table 3 shows the correlation coefficients between the histone amino acid residue distances from the interior of the dimers and their values of residue hydrophobicity for the histones of the *Gallus gallus *[1] (PDB id 2HIO) nucleosomal structure in the absence of DNA. One notes that the differential burial along the H3 chain is maintained as well as the distinction in values between species. It would be of interest to see if such differences are comparably maintained for the isolated dimers. If so, this would support the contention [8] that the difference in hydrophobic residue burial along the H3 histone chain is responsible for the difference in stability observed between the H3-H4 and H2A-H2B dimers in solution [9]. X-ray structures at atomic resolution have previously been obtained [29] for the individual homodimers of the A and B histones of the *Methanothermus fervidus *hyperthermophilic archaeon. In the presence of DNA, these dimers form tetramers [30] which are structurally homologous to the H3 and H4 eukaryotic nucleosomal tetramer.

Table 4 lists the correlation coefficients for the archaea. This includes results obtained for the x-ray structures [29] of the archaeal histones, HMfA (PDB id: 1B67) and HMfB (PDB id: 1A7W) of *Methanothermus Fervidus *While the difference in the correlation coefficients between residue distance and hydrophobicity from the n- to c-terminal regions of HMfA and HMfB is comparable with the difference from the n- to c-terminal regions of the eukaryotic H3 histones, the magnitude of burial over the homologous c-terminal regions is significantly less for the archaea. This difference in the burial of hydrophobic residues from the n- to c-terminal regions of the HMfA and HMfB archaeal chains can also be visually discerned from the asymmetry in values of the longer wavelength variations in hydrophobicity along the HMfA and HMfB sequences as seen in figure 8 of reference 8. Table 4 also lists the correlation coefficients for the histone chains of the HMk and HPhA hyperthermophiles. The HMk chain as well as the chain of HPhA show comparable differential burial of residues from the n- to c-terminal regions over their length as found for the methanogens, HMfA and HMfB.

**Figure 2:**
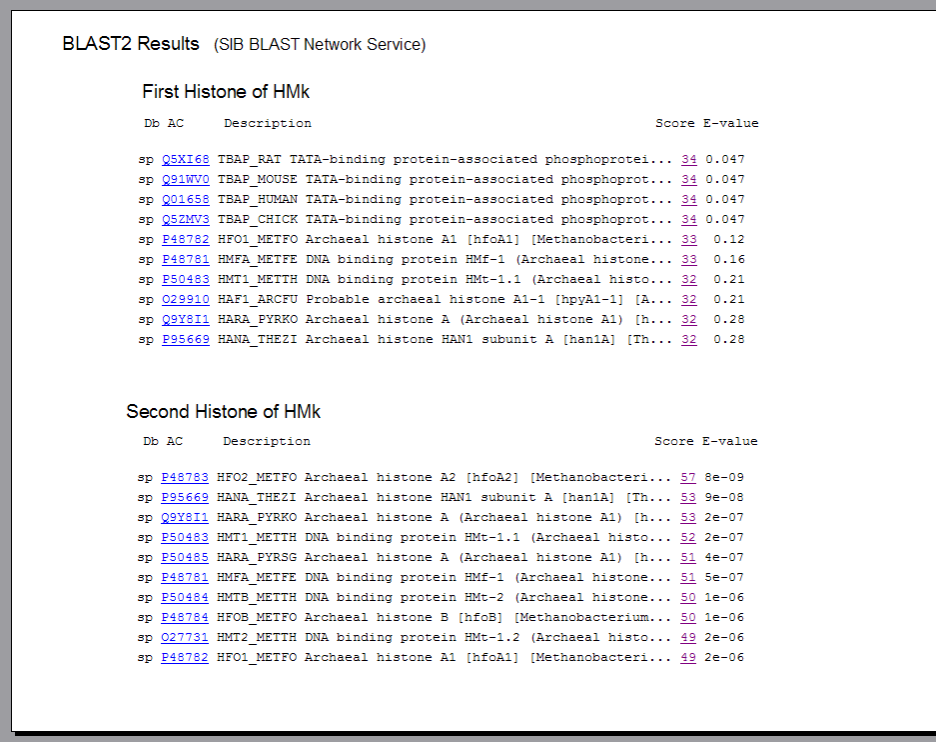
BLAST2 results for the individual first and second histones of HMk.

**Figure 3:**
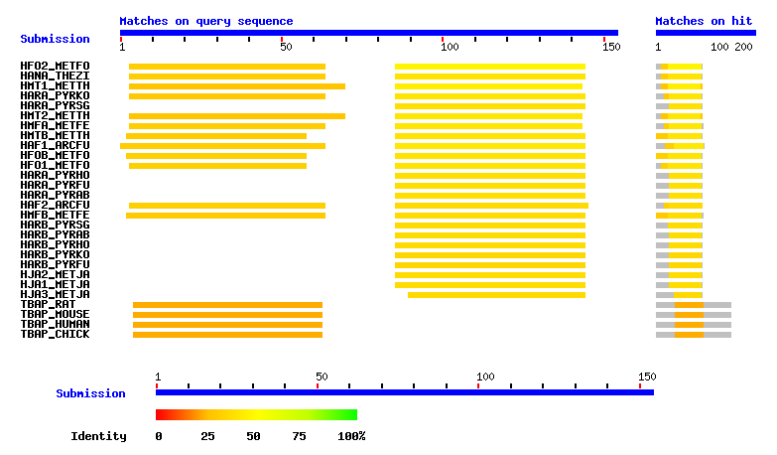
BLAST2 results for the entire sequence of the HMk doublet histone monomer.

HMk is particulary interesting for a number of reasons. First, the HMk monomer differs from the HMf and eukaryotic histones by containing two histone fold motifs in a single chain. Such anomaly is one of a number of strange properties exhibited by this archaeon [12,31,32]. Second, this monomeric doublet forms a dimer in the crystal [10] that appears to be structurally homologous to the (H3-H4)_2 _tetramer of the eukaryotic nucleosome. Gel filtration chromatography and chemical fixation have also indicated that in the absence of DNA the dominant form of HMk in solution is a stable dimer [33]. Furthermore, even though the two histones of HMk exhibit 28% sequence identity, they are exceedingly different in one respect. The first histone is distantly related to other known Archaeal sequences in the Swiss-Prot database, whereas the second histone has many close Archaeal neighbors in this database. Figure 2 shows the results of a BLAST2 search [23] of these two different histones. Interestingly, the closest hits of the first histone are four mammalian TATA box-binding proteins. The remaining Archaeal correspondences obtained are six-orders of magnitude greater in E-value than those obtained between the second histone and other Archaeal proteins in the data base. A PSI-BLAST search [22] reduces this difference to two-orders of magnitude; however, the four mammalian TATA box-binding proteins remain as nearest neighbors of the first histone. Figure 3 illustrates the segregated alignments of the two histones. The BLAST2 search [23] has been performed for the entire sequence of the doublet histone monomer. The color coded matches are shown along the full sequence. The four most distant correspondences of the ten shown are between the TATA box-binding proteins and the first histone.

**Figure 4:**
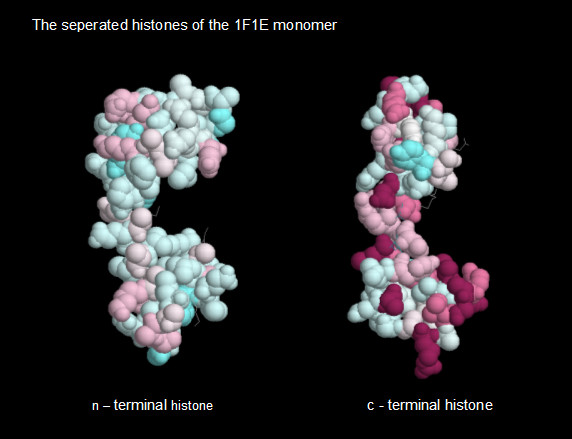
CONSURF coloring of the amino acid conservation of the histones of the HMk monomer.

Perhaps the most dramatic way to exhibit this difference between the two HMk histones is achieved by displaying the conservation of their amino acid residues. This is simply provided by a CONSURF analysis [25]. CONSURF accepts a PDB file (in the present case, the 1F1E PDB file), performs a PSI-BLAST sequence analysis and uses the results to assign an integer amino acid conservation score for each residue. The integer score is assigned a color which can be used to paint the atoms of the PDB structure and provide a visual display of amino acid conservation. The greatest integers, 9 (maroon) and 8 (magenta), are reserved for the most highly conserved residues. Figure 4 shows the two separate chains, the n- and c-terminal histones (first and second histones) of the 1F1E structure, painted with values obtained by an analysis of the full chain. Interestingly, whereas the c-terminal histone displays maroon and magenta, the n-terminal histone doesn't display any. The amino acid residues of the n-terminal histone have no 9's or 8's assigned, whereas the c-histone has been assigned 32, 9's and 8's.

Early analyses of the 16S rRNA sequence of HMk placed it close to the root of the Euryarchaeotic tree [31] while more recent studies based on more extensive sets of sequences [12,32] have grouped HMk with other archaeal methanogens. From this latter observation, *it thus comes as a surprise that its genome appears to contain large numbers of genes not present in the genomes of any of the other sequenced archaeal methanogens *and *it contains the largest fraction of genes for which function cannot automatically be assigned based on sequence similarity *[34]. Such equivocacy justifies speculation regarding the phylogeny of the HMk monomer within the context of two very different scenarios; one in which the HMk monomer evolved from a grouping with other methanogenic histones and one from a lineage in which it bore little resemblance to other archaeal histones.

**Figure 5:**
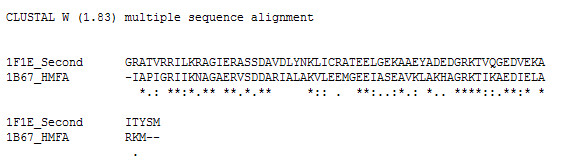
ClustalW alignment of the second HMk histone sequence with the archaeal HMfA sequence.

In the former scenario, the unique structural feature of HMk, namely, the tandem repeat of two histones in a single chain may have been the result of gene fusion. This, as well as other HMk unique protein fusions and splittings have been observed [12,31,32,34,35]. Reduced sequence homology between the first histone and other archaeal and eukaryotic H3 histones, from that observed with the HMf methanogens, may have resulted from gene duplication and subfunctionalization involving the n-terminal HMk histone. Relaxed constraints [13] combined with the selective pressures exerted by the extreme environment of the HMk organism could have enhanced tetramer stability at the cost of sequence homology with other methanogens. This is not inconsistent with the general observation that structurally related proteins need not be related in sequence. Significant modifications in sequence are, also, consistent with the high evolutionary rate inferred for this archaeon by a phylogenetic analysis of HMk's transcription apparatus [32]. Sequence homology between the c-terminal or second histone of HMk with the other archaeal histones would be relatively maintained. Figure 5 shows the close ClustalW alignment [36] between the sequence of the second histone and that of the methanogen, HMfA. The second HMk histone also aligns most closely with the H4 histones, which is consistent with H4's role of remaining constant throughout eukaryotic evolution.

For the second scenario, the ancestral HMk histones would have had little resemblance to other archaeal histones and differences between the first HMk histone and other archaeal histones would thereby be accounted for. Selection, however, would still have yielded the helices and structures required to wrap DNA and to bury the appropriate hydrophobic residues required to couple the monomers yielding structurally homologous dimers to the eukaryotic tetramers. Homology between the sequence of the second histone and other archaeal sequences as well as with the H4 eukaryotic histone would now require explanation. This homology could have been the result of lateral gene transfer (LGT). Such transfers have been found to occur; for example, the RNA polymerase subunit H of HMk has been apparently replaced by a protein from a distantly related protein of archaeal lineage [32]. This particular transfer is strongly supported by the observation of a well conserved insert of five or six amino acid residues shared only by the RNA polymerase subunits H from *M. kandleri *and *Thermoplasmatales*. Interestingly, the nearest BLAST2 [23] neighbor of the second histone of HMk is an archaeal histone of *Methanobacterium formicicum*. This histone and the second HMk histone share five identical amino acids that span the fold coupling the final two c-terminal histone helices. So, to summarize, either phylogenetic scenario would be consistent with the differences observed between the two histone sequences of HMk that had occurred during selection to enhance tetramer stability.

The HPhA and HMf archaea align with approximately 60% identity and their alignments with other archaeal histones are comparable. The differential asymmetry in the burial of hydrophobic residues from the n- to c-terminal ends of the HMk and HPhA chains is also comparable. For the HPhA dimers as for the HMf dimers, one might expect this bias to have been a consequence of selection that assisted in the coupling of the dimers to form tetramers while in the presence of DNA.

Table 5 lists the correlation coefficients for three histone-like dimers from the transcription factors of, TFIID [11], NF_Y [18] (PDB id; 1N1J), and NC2 [19] (PDB id 1JFI). The asymmetry of burial of hydrophobic residues from the n- to c-terminal region of the A chain of the 1TAF structure is comparable in magnitude with that found for the Archaea. This is consistent with the role played by this chain in binding the dimers of the tetramer. Such asymmetric burial is not observed for the B chain of the 1TAF structure. These observations are consistent with the homologies, originally identified [37,38] between the A and B chain sequences of the 1TAF PDB structure with the eukaryotic H3 and H4 histone sequences, respectively. As had been previously noted [18,19], the A and B chains of the histone-like structures of 1N1J and 1JFI more closely resemble the H2A and H2B histones than the H3, and H4 histones. Consequently differential burial along the chain lengths of these histone-like structures is not expected and the values of their correlation coefficients listed in Table 5 support this contention.

Major interest in the1TAF and 1F1E structures is a result of their apparent structural homology with the eukaryotic (H3-H4)_2 _tetramer. Both of these histones, however, differ in at least two respects. First, *Drosophila melanogaster*, DRm, is a member of the eukaryotic domain, whereas *Methanopyrus kandleri*, HMk, belongs to the archaeal domain and consequently at a greater phylogenetic distance from the eukaryotic structures. Secondly, the results of sequence alignments and BLAST2 [23] searches are very different for these two histone structures. The sequence of the A chain of 1TAF does resemble the nucleosomal H3 sequence and a BLAST2 [23] search, in contrast with the search of the first HMk sequence, turns up numerous related sequences. A detailed comparison of the apparently homologous HMk and DRm tetrameric structures is, therefore, of interest.

**Figure 6:**
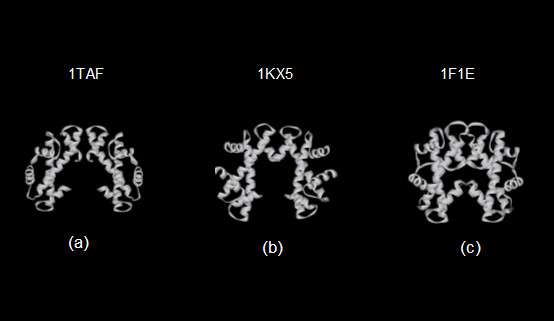
Ribbon diagrams of the homologous tetrameric histone structures from (a) *Drosophila melanogaster *(PDB id 1TAF) (b) *Xenopus laevis *(PDB id 1KX5) (c) *Methanopyrus kandleri *(PDB id 1F1E).

**Figure 7:**
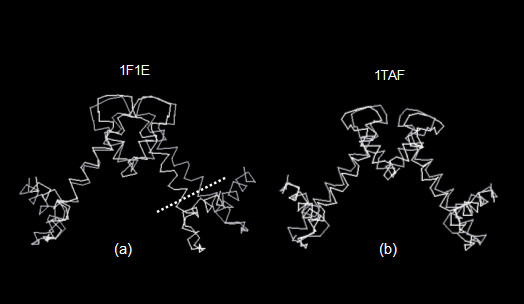
Combinatorial Extension (CE) alignment of the tetrameric histone structures of (a) *Methanopyrus kandleri *(PDB id 1F1E) and (b) *Drosophila melanogaster *(PDB id 1TAF) with *Xenopus laevis *(PDB id 1KX5).

Figure 6 shows ribbon diagrams of the three homologous tetrameric structures. They appear as inverted V-shaped shaped structures. From the figure it is seen that the arms of the V of HMk (figure 6c) are drawn together more closely than the arms of the eukaryotic structures (figures 6a and 6b). Closing the arms of the HMk structure places the c-terminal ends of the chains, homologous to the H4 eukaryotic chains, in close proximity. This proximity of the amino acids at the c-terminal ends of the HMk chains apparently contributes to the stability of the tetramer [10]. Figure 7 shows a wire diagram of the 1F1E and 1TAF α-carbon atom locations, CE [24] aligned with those of the eukaryotic 1KX5 structure. Only chains involved in the four-fold helical binding of the tetramer are shown. CE alignment is achieved by coupling the pairs of chains homologous to the H3 chains, as well as the H3 chains. Note that the alignment between the two eukaryotic structures is within the alignment threshold for the CE chain extension cut-off; consequently the chains are completely aligned. The RMSD between the alpha-carbon atom coordinates over the entire chain is 2.9 Angstroms with a sequence identity of 21 %. The CE alignment of the n-terminal histones of HMk with the H3 histones of 1KX5 is not possible over the entire length of the two arms of the structures, while still remaining below the CE threshold for chain extension. The longer length of HMk chain that is below this threshold is demarcated by the dashed line in figure 7. Over this aligned region, the RMSD is 2.7 Angstroms with a sequence identity of 8 %. Therefore, while the structure of the archaeal HMk dimer appears to be globally homologous with the tetrameric structure of the eukaryotic nucleosome and transcription factor, it exhibits well defined differences in structure as well as in sequence.

## Conclusion

The present investigation was predominantly motivated by the availability of structures at atomic resolution of the histone dimer of the archaeal hyperthermophile, HMk, and the histone-like tetramer of the eukaryotic transcription factor of *Drosophila melanogaster*, DRm. The asymmetry in burial of hydrophobic residues along the lengths of the histone chains of these structures was investigated as well as sequence and structural homology with other archaeal and eukaryotic, nucleosomal and transcription factor histones. Whereas previous studies have emphasized structural similarities between the HMk histone dimer and eukaryotic tetramers, the present study has emphasized differences in structure as well as in sequence.

The calculations show that the histone chains of the *Drosophila *transcription factor involved in the helical coupling that yield the tetramer, exhibit the asymmetry in the burial of hydrophobic residues previously observed for the homologous histone chains of the nucleosome. The magnitude of the burial over the c-terminal region of the A chain of the 1TAF structure is, however, less compared with that calculated for the chains of the nucleosomal proteins. On the other hand, all eukaryotic chains investigated that are not involved in such four-fold helical coupling, whether from the histone-like structures of transcription factors or from the histones of the nucleosome, do not exhibit this asymmetry. Consequently, while contributing to an instability or reduction in the binding of the lower order structures, the asymmetry may also provide a marker for the presence of higher order multimers currently unobserved.

The archaeal HMk histones have also been shown to exhibit the moderate asymmetry in residue burial comparable with that found for other archaeal histones and for the histones of the 1TAF structure. On the other hand, the histones of the HMk dimer that are involved in the four-fold helical coupling of the monomers, namely, the n-terminal histones of the monomer, have been shown to have questionable sequence and structural homology with archaeal and eukaryotic histones. The HMk histone not involved in such coupling, namely the c-terminal histone, is found to be homologous to numerous other archaeal histones in the Swiss-Prot database. How selection may have enhanced the stability of the tetramer by modifications that would be consistent with this difference between the two HMk histones has been described within the context of two different phylogenetic scenarios. The limited amount of data, however, makes these speculations tentative. It will be interesting to see how the story evolves as further structures are determined.

## Figures and Tables

**Table 1:** H3/H4 Eukaryotic Histones

		chain	amino half	carboxyl half	% change
1KX5	H3 A chain correlation coefficient	-0.363	-0.510	-0.242	-53
	H4 B chain correlation coefficient	-0.549	-0.596	-0.511	-14
1EQZ	H3 C chain correlation coefficient	-0.361	-0.470	-0.280	-40
	H4 D chain correlation coefficient	-0.550	-0.590	-0.517	12
1ID3	H3 A chain correlation coefficient	-0.437	-0.602	-0.293	-51
	H4 B chain correlation coefficient	-0.562	-0.639	-0.494	-22

**Table 2:** H2A/H2B Eukaryotic Histones

		chain	amino half	carboxyl half	% change
1KX5	H2A C chain correlation coefficient	-0.622	-0.662	-0.584	-12
	H2B D chain correlation coefficient	-0.571	-0.597	-0.531	-11
1EQZ	H2A A chain correlation coefficient	-0.617	-0.675	-0.546	-19
	H2B B chain correlation coefficient	-0.575	-0.600	-0.536	-11
1ID3	H2A C chain correlation coefficient	-0.619	-0.645	-0.600	-7
	H2B D chain correlation coefficient	-0.611	-0.662	-0.557	-16

**Table 3:** Eukaryotic Histones of 2HIO

	chain	amino half	carboxyl half
H3 C chain correlation coefficient	-0.389	-0.458	-0.276
H4 D chain correlation coefficient	-0.569	-0.618	-0.559
H2A A chain correlation coefficient	-0.558	-0.587	-0.546
H2B B chain correlation coefficient	-0.542	-0.580	-0.545

**Table 4:** Archaeal Histones

		chain	amino half	carboxyl half	% change
1B67	correlation coefficient	-0.516	-0.641	-0.390	-39
1A7W	correlation coefficient	-0.537	-0.633	-0.443	-30
1F1E	First histone (A chain) correlation coefficient	-0.600	-0.772	-0.437	-43
	Second Histone (A chain) correlation coefficient	-0.561	-0.637	-0.464	-27
1KU5	correlation coefficient	-0.539	-0.695	-0.446	-36

**Table 5:** Transcription Factor Histones

		chain	amino half	carboxyl half	% change
1TAF	dTAF42 A chain correlation coefficient	-0.506	-0.610	-0.398	-34
	dTAF62 B chain correlation coefficient	-0.460	-0.458	-0.491	7
1N1J	A chain correlation coefficient	-0.595	-0.585	-0.620	5
	B chain correlation coefficient	-0.523	-0.473	-0.571	21
1JFI	A chain correlation coefficient	-0.657	-0.642	-0.686	7
	B chain correlation coefficient	-0.663	-0.658	-0.670	2
